# In-depth analysis of immune cell landscapes reveals differences between lung adenocarcinoma and lung squamous cell carcinoma

**DOI:** 10.3389/fonc.2024.1338634

**Published:** 2024-01-25

**Authors:** Xinfeng Wang, Keao Zheng, Zhiying Hao

**Affiliations:** Department of Pharmacy, Shanxi Province Cancer Hospital/Shanxi Hospital Affiliated to Cancer Hospital, Chinese Academy of Medical Sciences/Cancer Hospital Affiliated to Shanxi Medical University, Taiyuan, Shanxi, China

**Keywords:** lung adenocarcinoma, squamous cell carcinoma, biomarkers, digital cytometry, machine learning

## Abstract

**Background:**

Lung cancer is the leading cause of cancer deaths globally, with lung adenocarcinoma (LUAD) and squamous cell carcinoma (LUSC) being major subtypes. Immunotherapy has emerged as a promising approach for the treatment of lung cancer, but understanding the underlying mechanisms of immune dysregulation is crucial for the development of effective therapies. This study aimed to investigate the distinctive cellular features of LUAD and LUSC and identify potential biomarkers associated with the pathogenesis and clinical outcomes of each subtype.

**Methods:**

We used digital cytometry techniques to analyze the RNA-Seq data of 1128 lung cancer patients from The Cancer Genome Atlas (TCGA) database. The abundance of cell subtypes and ecotypes in LUAD and LUSC patients was quantified. Univariate survival analysis was used to investigate their associations with patient overall survival (OS). Differential gene expression analysis and gene co-expression network construction were carried out to explore the gene expression patterns of LUSC patients with distinct survival outcomes. Scratch wound-healing assay, colony formation assay, and transwell assay were used to validate the candidate drugs for LUSC treatment.

**Results:**

We found differential expression of cell subtypes between LUAD and LUSC, with certain cell subtypes being prognostic for survival in both subtypes. We also identified differential gene expression and gene co-expression modules associated with macrophages.3/PCs.2 ratio in LUSC patients with distinct survival outcomes. Furthermore, ecotype ratios were found to be prognostic in both subtypes and machine learning models showed that certain cell subtypes, such as epithelial.cells.1, epithelial.cells.5, and endothelial.cells.2 are important for predicting LUSC. Ginkgolide B and triamterene can inhibit the proliferation, invasion, and migration of LUSC cell lines.

**Conclusion:**

We provide insight into the distinctive cellular features of LUAD and LUSC, and identify potential biomarkers associated with the pathogenesis and clinical outcomes of each subtype. Ginkgolide B and triamterene could be promising drugs for LUSC treatment.

## Introduction

Lung cancer is one of the most common and deadliest cancers worldwide (1). With an estimated 2.2 million new cases and 1.8 million deaths in 2020, lung cancer is currently the leading cause of cancer deaths globally (2). Non-small-cell lung cancer (NSCLC) accounts for approximately 85% of all cases (3). Among the different types of NSCLC, lung adenocarcinoma (LUAD) and squamous cell carcinoma (LUSC) are the two major histological subtypes (3). LUAD is more prevalent among non-smokers, while LUSC is strongly associated with a history of smoking, particularly among current or former heavy smokers. Despite sharing a common origin in the lung, LUAD and LUSC differ in their biological and clinical characteristics, including their molecular profiles, cell origins, histological features, prognosis, and responses to treatment (3). LUAD mainly arises from the glandular cells and has a glandular or acinar structure. LUSC originates from the squamous cells lining the airways and appears as sheets of flat cells. LUAD component could transform to LUSC by transdifferentiation (4). Common genetic alterations include mutations in EGFR, KRAS, BRAF, and P53 genes, which are important targets for drug therapy and prognosis (5). The survival rate for LUAD is generally higher than for LUSC (6). Compared to LUAD patients, individuals with LUSC exhibited a higher prevalence of symptoms such as cough, fever, and abundant sputum, and a greater incidence of bacterial and fungal infections (3). The efficacy of targeted therapies, such as immune checkpoint inhibitors and EGFR and BRAF inhibitors in LUAD, has been well established (5). However, there is limited availability of targeted therapies for LUSC, and the treatment often involves surgery, radiation, and platinum-based chemotherapy (7). Therefore, understanding the key differences between these two subtypes and their underlying mechanisms is critical for developing effective personalized therapies and improving patient outcomes.

In lung cancer, immune dysregulation is a prominent feature (8). Immune cells within the tumor microenvironment can play a dual role in promoting and inhibiting tumor growth, and the imbalance between these opposing forces can impact the outcome of the disease. Immunotherapy has emerged as a promising approach for the treatment of lung cancer in recent years, and understanding the underlying mechanisms of immune dysregulation in this disease is crucial for the development of effective therapies (9). Therefore, an in-depth investigation of the role of immune dysregulation in lung cancer has significant clinical implications.

Digital cytometry techniques have emerged as a complementary tool to scRNA-seq, which necessitates using antibodies for physically isolating tissue cells from fresh specimens (10). Ecotyper, based on CIBERSORTx, holds advantages such as identifying cell states, estimating their relative abundance in each sample, retrieving them in external expression datasets, and discovering co-association patterns between cell states that make up the cancer ecosystems (11). Currently, few studies investigated the differences between the two non-small cell lung cancer (NSCLC) subtypes LUAD and LUSC (8, 9, 12). The cell subtypes, cell ratios, and ecotypes have not been comprehensively investigated in LUAD and LUSC. Therefore, we aim to analyze the prognostic value of cell subtypes, cell ratios, and ecotypes in LUAD and LUSC survival.

**Figure 1:**
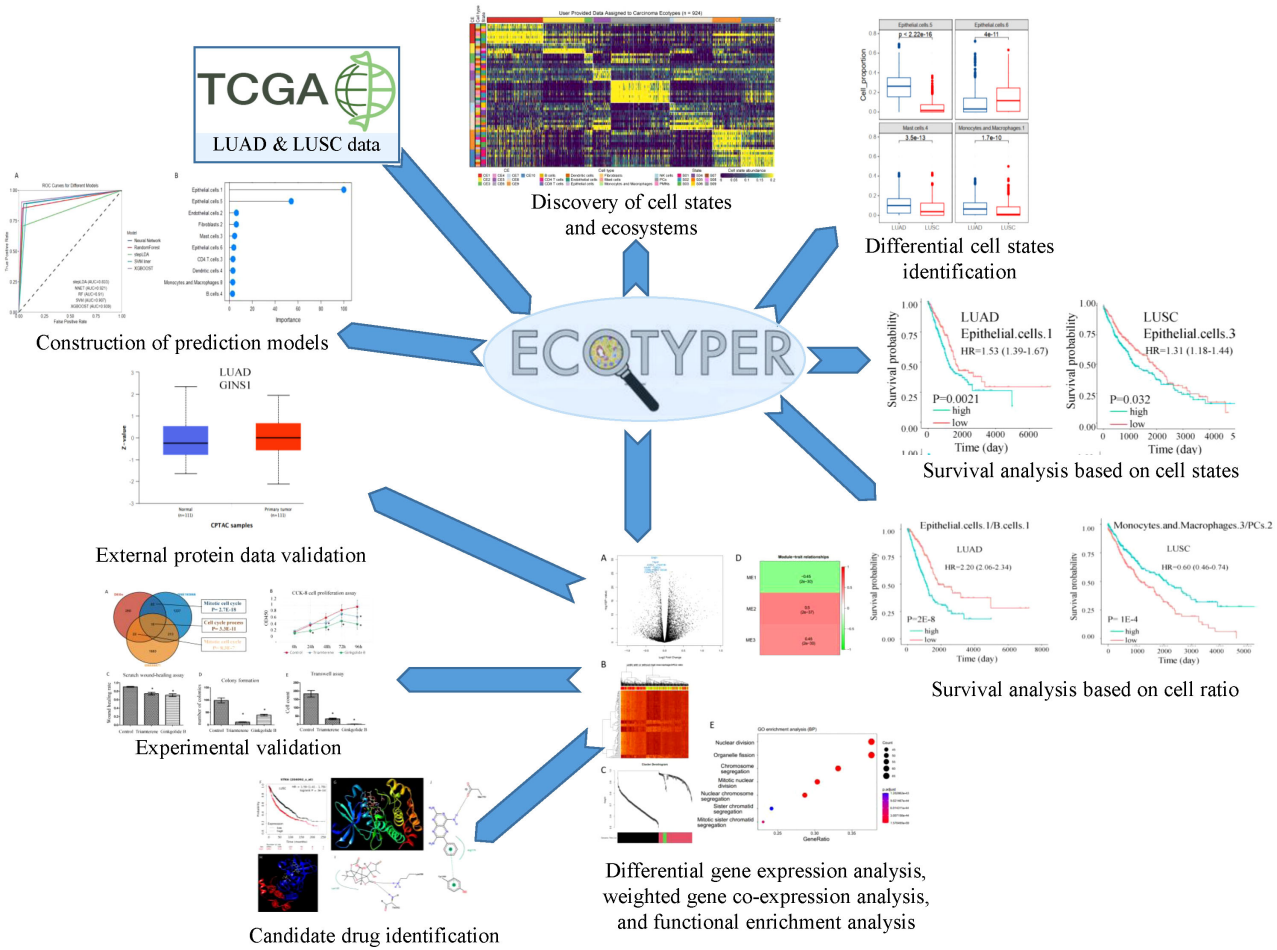
A schematic diagram illustrates the study workflow.

In this study, we aim to investigate and compare the distinctive clinical and molecular features of LUAD and LUSC, and to identify potential biomarkers associated with the pathogenesis and clinical outcomes of each subtype. The analysis framework is shown in **Figure 1**. Our analysis could offer valuable insights that might be useful in real-world medical applications.

## Materials and methods

### Dataset preparation

Lung cancer RNA-Seq and survival data were downloaded from the TCGA database at https://portal.gdc.cancer.gov/. A total of 1128 lung cancer patients from TCGA were used for analysis. The expression matrix with gene symbols as the first column was uploaded to the online Ecotyper tool at https://ecotyper.stanford.edu/carcinoma/ (11). The resulting files for cell states and ecotypes were downloaded and merged into one file for downstream analysis. Protein expression data were extracted from the UALCAN database (https://ualcan.path.uab.edu/) (13, 14). Two additional datasets were downloaded from the NCBI GEO database for drug treatment validation. GSE85871 contains gene expression profiles of MCF7 cells treated with 10 μM Ginkgolide B (two replicates) and DMSO (six replicates) (15). GSE193855 contains gene expression profiles of diffuse intrinsic pontine glioma cells treated with triamterene (three replicates) and DMSO (three replicates) (16). GSE154286 contains profiles of a panel of 201 genes from NSCLC patient biopsies before and after platinum-based chemotherapy (7).

### Survival analysis

The associations of cell subtypes, subtype ratios, ecotypes, ecotype ratios, or gene expression with patient overall survival (OS) were analyzed by univariate survival analysis in the R survival package. Multivariate analysis was used to validate the significance with adjustment for smoking or copy number alterations of BRAF, EGFR, KRAS, and P53. The patients were divided into two groups split at the median values of independent variables. Kaplan-Meier survival curves were plotted by ggsurvplot in the R survminer package. The significance of the survival rate difference between the two groups was calculated by the log-rank test. Unless otherwise stated, a P value of less than 0.05 was considered significant. Survival plots for cell subtype marker genes were derived from the Kaplan-Meier plotter database (https://kmplot.com/) (17).

### Differential gene expression analysis and gene co-expression network construction

The differential gene expression in macrophages.3/PCs.2-high samples and other samples were analyzed by the R limma package. The high-ratio samples had longer survival and were treated as control. The log_2_(fold change) and P values were set as 0.5 and 0.05 and the P value adjustment method is “BH”. For gene co-expression module identification, the differential expression genes matrix was analyzed according to manuals (18, 19). The parameters were set as softPower =14, corFnc = “cor”, Networktype = “signed”, minModuleSize = 10, deepSplit = 4. For module functional annotation, clusterProfiler was used to get the significant terms and related bubble plots (20).

### Scratch wound-healing assay, colony formation assay, and transwell assay

LUSC cell line HCC95 was obtained from the American Type Culture Collection (ATCC) and was cultured in RPMI-1640 medium (Keygen Biotech, China) supplemented with 10% fetal bovine serum (FBS, Gibco, Grand Island, USA). The cells were maintained in a humidified incubator at 37°C with 5% CO_2_. Ginkgolide B (CAS No. 15291-76-6, BN52022) was acquired from Tauto Biotech Co., Ltd. (Shanghai, China) and triamterene (CAS No. 396-01-0, T4143) was purchased from Sigma-Aldrich. We did a preliminary experiment to find a proper concentration for treatment. We used the two concentrations 100 mg/L for triamterene and 200 mg/L for ginkgolide B as they approached the half-maximal inhibitory concentration (IC50) at 96 h. Therefore, the following assays used the two concentrations.

The scratch wound-healing assay, colony formation assay, and transwell assay were employed to investigate the impact of Ginkgolide B and triamterene on the survival, invasion, and migration of LUSC cell line HCC95. The scratch wound-healing assay provides insights into cell migration dynamics, the colony formation assay evaluates long-term proliferation capacity, and the transwell assay measures invasion potential. Moreover, the choice of these assays aligns with the need for a comprehensive understanding of the effects of the tested compounds on different facets of cancer cell behavior.

To assess cell survival, the cells were seeded in a 96-well plate at a density of 3×10^3^ cells per well with 200 mg/L Ginkgolide B, 100 mg/L triamterene or DMSO (n= 5 each group). The cells were subsequently incubated at 37°C in a medium containing 10% Cell Counting Kit-8 (CCK-8; Dojindo Inc., Kumamoto, Japan). Survival rates were assessed at 0, 24, 48, 72, and 96 hours by measuring the absorbance at 450 nm.

For the invasion assay, Transwell Matrigel invasion chambers in two 24-well plates (pore size, 8 µm; BD Biosciences, San Jose, CA, USA) were used according to the published procedure (21). Briefly, the cells were serum-starved for 6 h in RPMI-1640 containing 0.1% FBS. Serum-starved cells were trypsinized and resuspended in RPMI-1640 containing 0.1% FBS, and 200 µL serum-free medium containing 3×10^5^ cells from each subgroup was added to the upper chamber of each well coated with 50 mg/L Matrigel (BD Biosciences). A volume of 0.6 mL 15% FBS-containing medium was then added to the lower chamber as a chemoattractant. After 24 h at 37°C, the cells on the upper membrane surface were removed with a cotton swab. The inserts were fixed by treatment with 95% ethanol for 30 min and stained with 0.1% crystal violet solution (Beyotime Institute of Biotechnology, Shanghai, China) at 37°C for 30 min. The cells on the bottom of the membrane were counted from three different light microscopic fields, and the mean number of cells was calculated.

Anchorage-dependent (liquid) colony formation assays were conducted according to the published procedure (22). Briefly, 3×10^2^ cells were seeded in each well of a 6-well plate and cultured in RPMI-1640 medium supplemented with 10% FBS and with 200 mg/L Ginkgolide B, 100 mg/L triamterene or DMSO for 10 days. Cells were washed twice with phosphate buffered saline (PBS), then fixed with 4% paraformaldehyde in PBS, and stained with 200 uL 0.1% sulforhodamine-B (Sigma) dissolved in 1% acetic acid for 30 min at room temperature. The dye was aspirated, and the wells were washed three times with 1% acetic acid to remove unbound stain. No media change was performed during the assay.

In the scratch wound-healing assay, a total of 3×10^5^ cells were seeded into RPMI-1640 medium supplemented with 10% FBS in a 6-well tissue culture plate. After 48 hours, the cell monolayer reached approximately 80% confluence. Subsequently, a straight line scratch was created in one direction using a 20-µL pipette tip, gently disrupting the cell monolayer. The well was then washed twice with PBS to remove any detached cells. Fresh medium with 200 mg/L Ginkgolide B, 100 mg/L triamterene, or DMSO was added to each well to replace the existing medium. Wound healing was monitored by capturing images of the scratch at 0, 24, and 48 hours post-wounding using an inverted light microscope with the same settings. Three random representative images were taken for each time point. The wound area was quantitatively analyzed using ImageJ software by measuring the blank area in the images.

### Protein docking

For drug screening, up-regulated differential genes were submitted to the Connectivity Map, and significant results were determined at a significance level of P < 0.01 (23). Protein-ligand docking analysis was executed using SwissDock (24). For visualizing the interactions between the protein and ligand, LigPlot+ was employed to generate a pose view (25).

### Statistical analysis

The statistical significance of the mean difference between the two groups was calculated by the t-test. In all statistical analyses, a P value of less than 0.05 was considered significant. Cell states abundance matrix was input into R for machine learning prediction. In the R caret package, the 71 cell states abundance matrix of 1128 lung cancer samples were used to predict disease type LUAD or LUSC. Stratified random sampling was used to divide samples into 75% for training and 25% for validation. Five popular machine learning methods were used including support vector machines (svmLinear), feed-forward neural networks (nnet), extreme gradient boosting (xgbTree), random forests (ranger), and linear discriminant analysis with stepwise feature selection (stepLDA) with default parameters. The differentially expressed genes between the 31 paired patients of pre- and post-platinum-based chemotherapy in GSE154286 were identified by paired t test.

## Results

### Cell subtypes are differentially expressed between LUAD and LUSC

**Figure 2:**
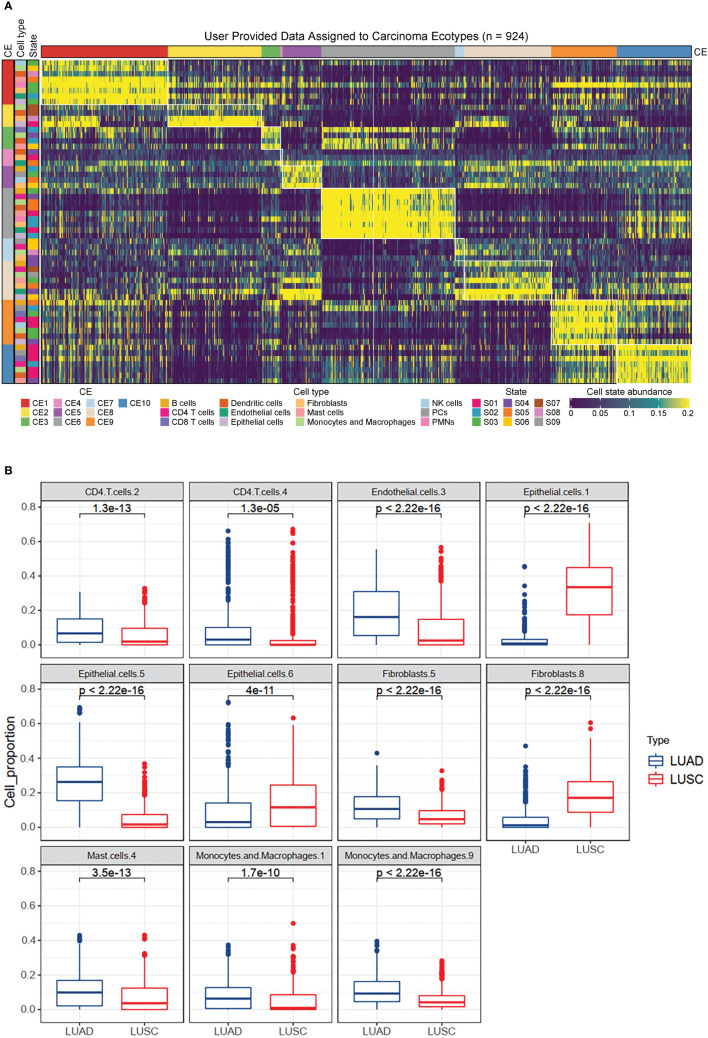
Immune landscapes in lung cancer. **(A)** Heatmap depicting ten carcinoma ecotypes (CE) and 72 cell states identified from digitally purified lung cancer transcriptomes. Patient samples (columns) are organized into CE clusters. **(B)** Box plots showing the differential expression of eleven cell subtypes between LUAD and LUSC patients. The analysis was based on 1128 lung cancer patients from the TCGA database.

Ecotyper, a digital cytometry method, was used to identify cell subtypes based on bulk transcriptome data and to quantify their relative abundance in each sample (11). Key marker genes for the cell subtypes are provided in **Supplementary Table 1**. A total of 72 cell subtypes from 16 cell types were quantified (**Supplementary Table 2**). These cell subtypes were assigned to 10 higher-level carcinoma ecotypes (**Figure 2A**; **Supplementary Table 3**). Differential analysis revealed the differences in cell subtypes between LUAD and LUSC (**Supplementary Table 4**). For example, CD4.T.cells.2, endothelial.cells.3, epithelial.cells.5, fibroblasts.5, mast.cells.4, macropahges.1, and macropahges.9 were significantly down-regulated in LUSC compared to LUAD (**Figure 2B**). Epithelial.cells.1, epithelial.cells.6, and fibroblasts.8 were significantly up-regulated in LUSC (**Figure 2B**). According to Ecotyper, the annotation of the 11 differential cell subtypes is listed in **Table 1**.

**Table 1:** Annotation and marker gene for the differential expressed cell subtypes.

Cell subtypes	Annotation (Marker gene)	Cell subtypes	Annotation (Marker gene)
CD4.T.cells.2	Naïve/central memory (CCR7)	Fibroblasts.5	Unknown (PPP1R32)
CD4.T.cells.4	Resting (KLF2)	Fibroblasts.8	Pro-migratory-like (CA9)
Endothelial.cells.3	Unknown	Mast.cells.4	Classical (TPSAB1)
Epithelial.cells.1	Basal-like (K6C)	Macropahges.1	Monocytes (CCR2)
Epithelial.cells.3	Pro-angiogenic (ITGA3)	Macropahges.9	Unknown (DLEC1)
Epithelial.cells.5	Unknown (AGR2)		
Epithelial.cells.6	Metabolic (HP1-BETA)		

### Differentially expressed cell subtypes are prognostic for survival in LUAD and LUSC

**Figure 3:**
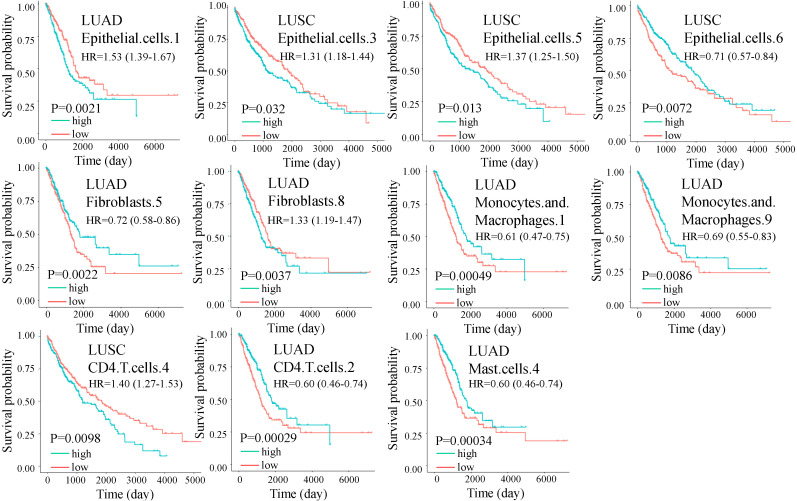
Overall survival (OS) for cell subtypes in LUAD and LUSC. The green and red lines indicate high and low expression of immune cells, respectively. Significance was determined by a two-sided log-rank test. In multivariate analysis, all the cell subtypes were still significant after adjusting for copy number alterations of BRAF, EGFR, KRAS, and P53 except fibroblasts.8.

The prognostic impact of cell subtypes in LUAD and LUSC has not been fully explored, we used the abundance matrix of cell subtypes to calculate their associations with patient survival. We found that 10 of the 11 differential cell subtypes were significantly associated with overall survival (**Figure 3**). CD4.T.cells.2 had the most significant prognostic ability, which had a favorable effect on survival, while CD4.T.cells.4 had an adverse prognosis (**Figure 3**). All the cell subtypes were still significant after adjusting for copy number alterations of BRAF, EGFR, KRAS, and P53 except fibroblasts.8.

### Marker genes of cell subtypes are prognostic of survival in lung cancer

**Figure 4:**
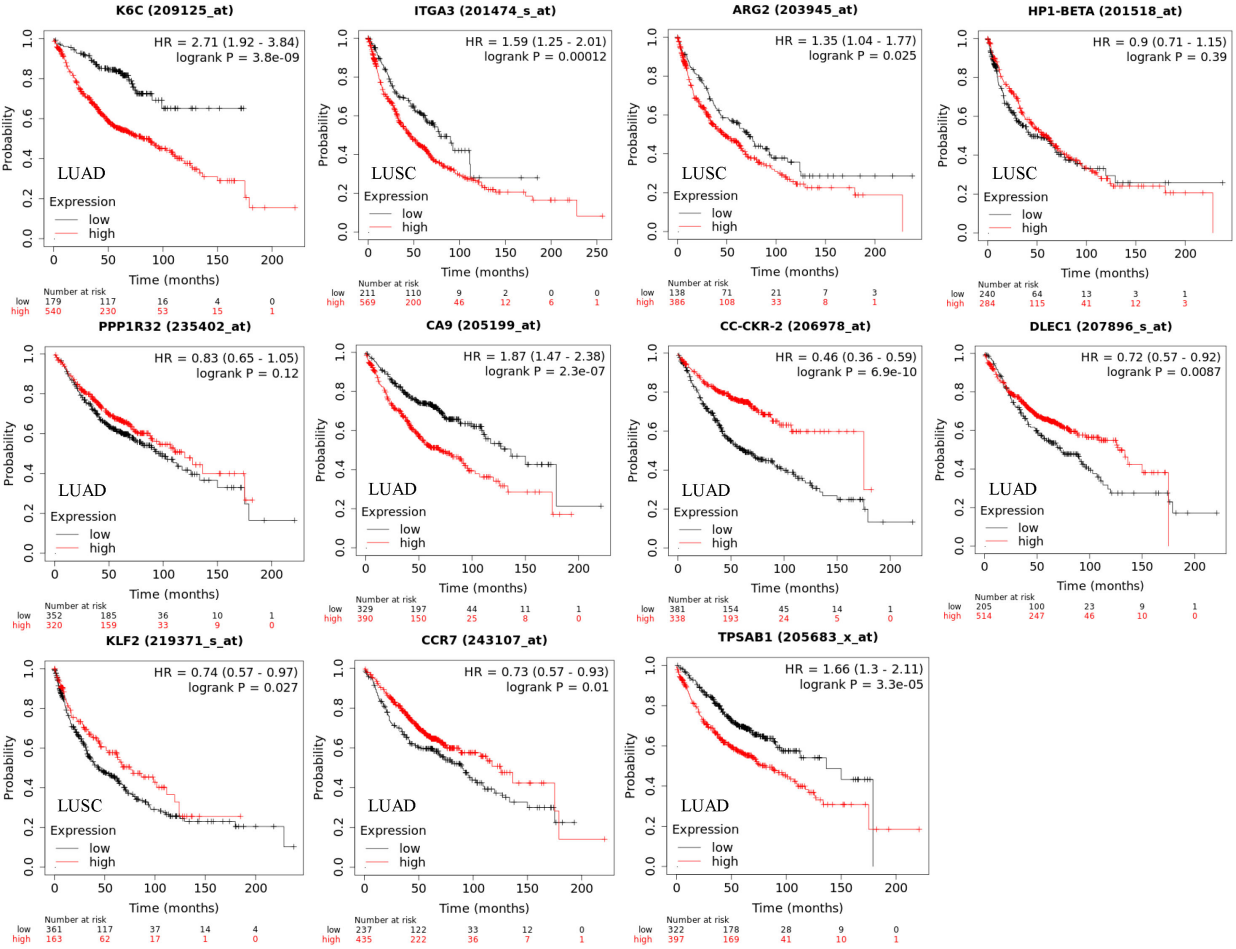
Overall survival (OS) for cell subtype marker genes in LUAD and LUSC corresponding to **Figure 2**. The marker genes for cell states are KRT6A (epithelial.cells.1), ITGA3 (epithelial.cells.3), ARG2 (epithelial.cells.5), HP1-BETA (epithelial.cells.6), PPP1R32 (fibroblasts.5), CA9 (fibroblasts.8), CC-CKR-2 (monocytes.and.macrophages.1), DLEC1 (monocytes.and.macrophages.9), KLF2 (CD4.T.cells.4), CCR7 (CD4.T.cells.2), and TPSAB1 (mast.cells.4). Significance was determined by a two-sided log-rank test. Genes PP1R32 and CCR7 were not significant after adjusting for smoking.

We validated the clinical relevance of cell subtypes by checking the prognostic value of marker genes of each cell subtype. We found that all the marker genes were prognostic for patient survival (**Figure 4**). For example. K6C, a marker of epithelial.cells.1, is an adverse gene in the LUAD. ARG2, a marker of epithelial.cells.5, is an adverse gene in the LUSC. CA9, a marker of fibroblasts.8, is an adverse gene in the LUAD. CCR2, a marker of macrophages.1, is a favorable gene in the LUAD. However, genes PP1R32 and CCR7 were not significant after adjusting for smoking.

### Ratios of cell subtype are prognostic in lung cancer

**Figure 5:**
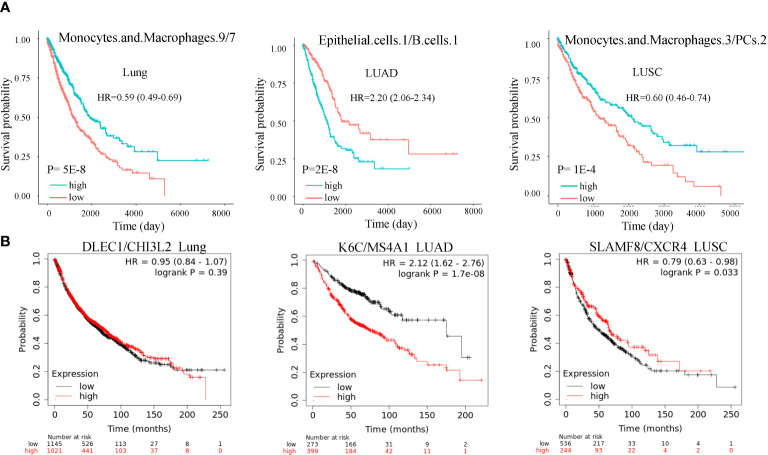
Cell subtype ratios and validation in lung cancer. **(A)** Cell subtype ratios of macrophages.9/macrophages.7, epithelial.cells.1/B.cell.1, and macrophages.3/PCs.2 are predictive of lung cancer survival. These cell subtype ratios were still significant after adjusting for copy number alterations of BRAF, EGFR, KRAS, and P53. **(B)** Validation of cell subtype ratios by the marker gene ratios in the independent dataset. Ratios K6C/MS4A1 and SLAMF8/CXCR4 were still significant after adjusting for smoking.

As mentioned previously, the cell state is heterogeneous. We questioned if ratios of different cell subtypes could also prognosis. We found hundreds of cell ratios that can significantly separate patients with high and low survival rates. For example, macrophages.9/macrophages.7, epithelial.cells.1/B.cells.1, and macrophages.3/PCs.2 were the most significantly associated with survival in lung cancer, LUAD, and LUSC (**Figures 5A**). Macrophages.3 and macrophages.7 were annotated as Classical M1 and M2-like proliferative macrophage subsets. B.cells.1 was annotated as a classical naïve subset. PCs.2 was annotated as an unknown subset. In an independent dataset (17), we validated the cell ratios by the cell subtype marker gene ratios and confirmed their roles in OS (**Figure 5B**). These results were significant after adjusting for smoking and genetic background.

### Differential gene expression analysis in LUSC patients with a high and low ratio of macrophages.3/PCs.2

**Figure 6:**
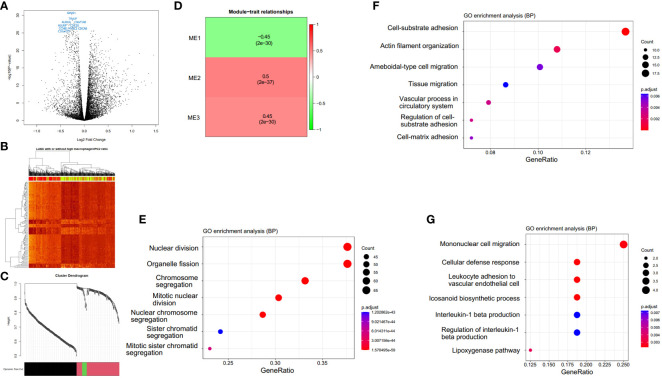
Macrophages.3/PCs.2-high expression samples have distinct expression patterns. **(A)** A total of 392 differential expression genes (DEGs) are identified. **(B)** The clustering heat map based on DEGs shows the distinct expression patterns between groups. In the color bar above the heat map, yellow indicates macrophages.3/PCs.2-low expression samples, while red indicates macrophages.3/PCs.2-high expression samples. **(C)** The cluster dendrogram shows the assignment of genes into gene modules with different colors. The color bar indicates the assignment of genes to a module. **(D)** Module trait relationship heatmap indicates the correlation between module expression and macrophages.3/PCs.2 ratio. GO biological process enrichment and of module Mod1 **(E)**, Mod2 **(F)**, and Mod3 **(G)** genes by clusterProfiler.

**Figure 7:**
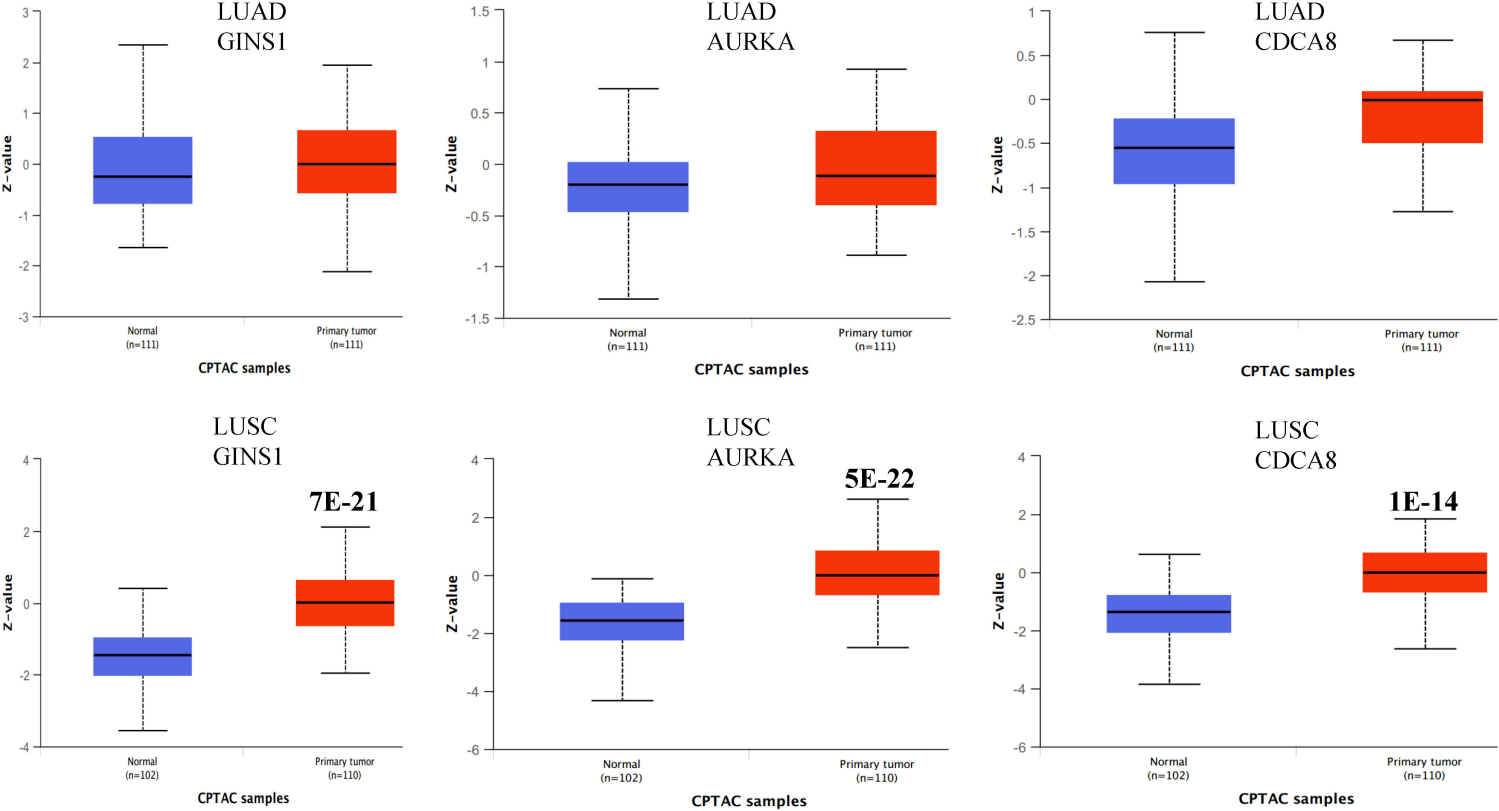
Boxplots showing the protein expression of three differential genes GINS1, AURKA, and CDCA8 in normal lung, LUAD, and LUSC. The P values above the box indicate the significance. No P value indicates no significance between the two groups. The protein expression data was extracted from the UALCAN database.

We explored the gene expression patterns of the two groups of LUSC patients with distinct survival outcomes identified by the expression of macrophages.3/PCs.2. The differential expression genes (DEGs) were identified by comparing samples with low and high ratios of macrophages.3/PCs.2. A total of 392 DEGs were identified (**Figure 6A**). The 10 most significant genes include GINS1, TRAIP, AURKA, C9orf140, HJURP, CDC20, CCNB1, MYBL2, CDCA8, and C16orf75, all of which were down-regulated in macrophages.3/PCs.2 low expression samples. All of the 10 genes, except HJURP, were identified as unfavorable prognostic factors in LUAD. In LUSC, HJURP, CDC20, CCNB1, and MYBL2 were unfavorable prognostic factors, while GINS1 and C16orf75 were identified as favorable prognostic factors (data not shown). Cluster analysis based on DEGs showed that macrophages.3/PCs.2 low expression samples were under the same major branch (**Figure 6B**). We performed gene co-expression analysis to check if these genes are organized into functional modules. Only three modules were identified with distinct biological functions and all of the modules were significantly associated with macrophages.3/PCs.2 ratio (**Figures 6C, D**). Mod1 was mainly associated with nuclear division and cell cycle (**Figure 6E**). Mod2 was associated with cell-substrate adhesion (**Figure 6F**). Mod3 was associated with macrophage migration (**Figure 6G**). We confirmed three of the significant differential genes GINS1, AURKA, and CDCA8 at the protein level (**Figure 7**).

### Cell subtypes are organized into ecotypes, and ecotype ratios are prognostic in lung cancer

**Figure 8:**
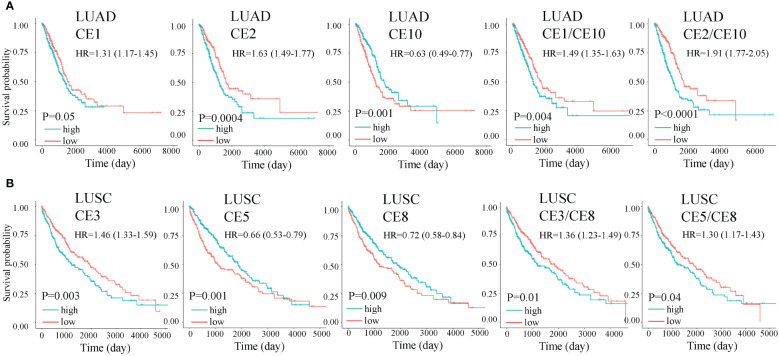
Overall survival (OS) for carcinoma ecotypes (CEs) and CE ratios in LUAD **(A)** and LUSC **(B)**. Significance was determined by a two-sided log-rank test. In multivariate analysis, all the cell ecotypes and ecotype ratios were still significant after adjusting for copy number alterations of BRAF, EGFR, KRAS, and P53 except CE5/CE8 (P= 0.055).

It has been found that cell subtype abundance profiles across 16 carcinomas could organize into ten carcinoma ecotypes (CEs) (11). We questioned if CEs and ratios of different CEs could also be prognostic. We found six CEs and hundreds of CE ratios that can significantly separate patients with high and low survival rates. For example, CE1, CE2, and CE10 were prognostic in LUAD, while CE3, CE5, and CE8 were prognostic in LUSC (**Figure 8**). Interestingly, we found that CE2/CE10 had a smaller P value than CE1 and CE10 in LUAD (**Figure 8A**). The ratio of prognostic CEs was still prognostic in LUSC, such as CE3/CE8 and CE5/CE8 (**Figure 8B**).

### Machine learning models to predict LUAD and LUSC based on cell subtypes

**Figure 9:**
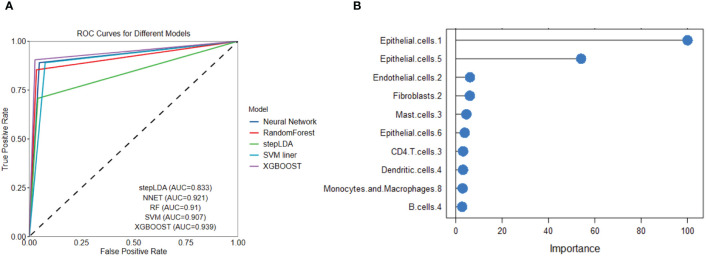
Five popular machine learning models were used to predict LUAD and LUSC based on cell subtype abundance. **(A)** ROC curves for the five machine learning models show that XGBOOST had the best LUSC prediction performance. **(B)** Variable importance analysis reveals the top 10 most significant cell subtypes in LUSC prediction.

We used several popular machine learning models to predict LUAD and LUSC using the cell subtype profiles. Among the five popular methods, we found XGBOOST had the highest performance in separating LUSC from LUAD (**Figure 9A**). The variable importance analysis also indicated the cell subtype differences between LUAD and LUSC (**Figure 9B**). Epithelial.cells.1, epithelial.cells.5, endothelial.cells.2, and fibroblasts.2 were among the top contributing variables in more than two machine learning models. Endothelial.cell.2 was annotated as tip-like ECs. Fibroblasts.2 was annotated as normal enriched fibroblasts.

### Candidate drugs screening based on differentially expressed genes

**Figure 10:**
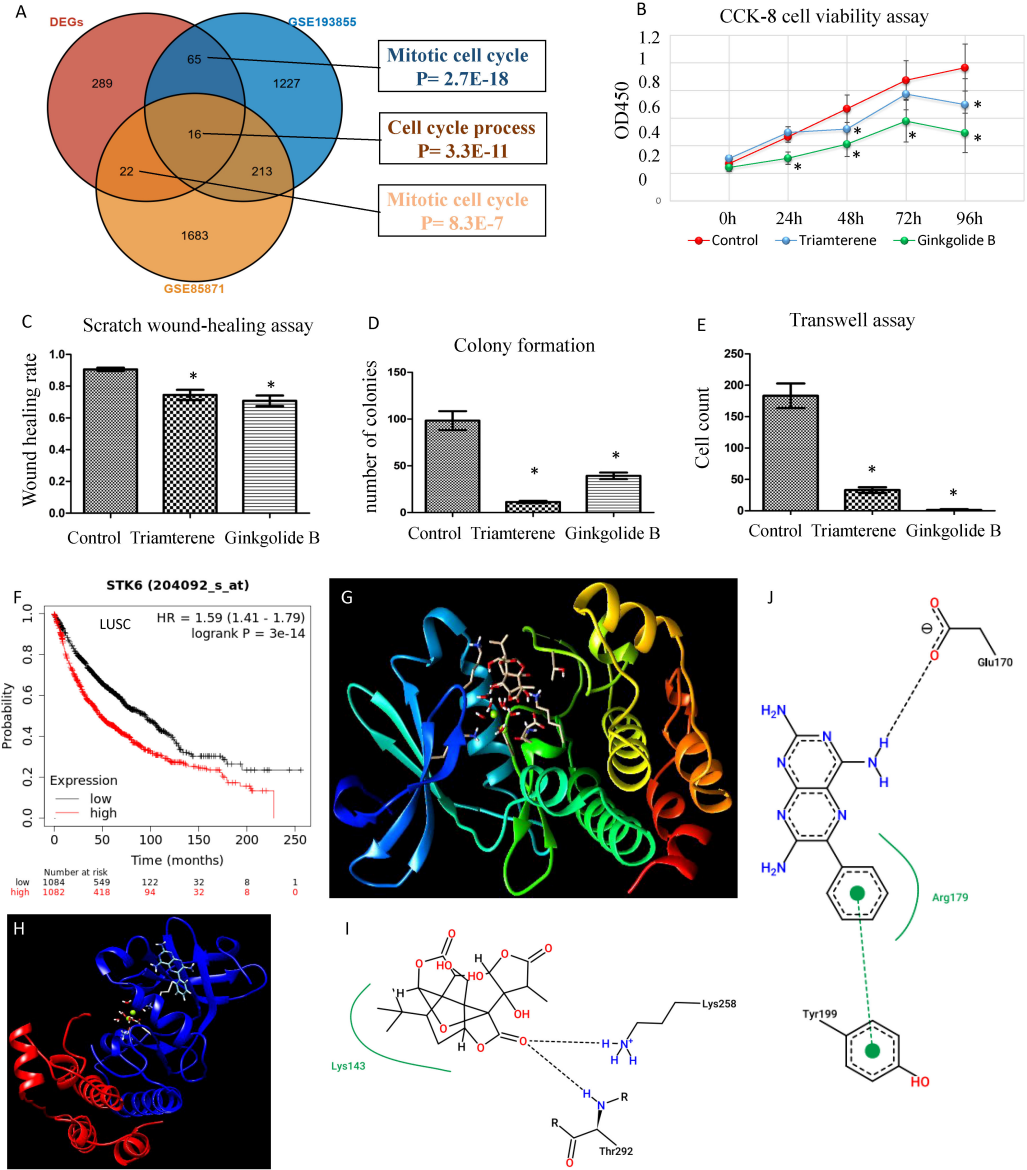
Validation of candidate drugs for LUSC treatment. **(A)** Validation of two candidate drugs ginkgolide B and triamterene in two independent datasets GSE85871 and GSE193855. Gene overlap of three gene lists. DEGs: differential genes of samples with high and low ratios of macrophages.3/PCs.2. **(B)** Ginkgolide B and triamterene treatment of the HCC95 cell line inhibit cell survival (n= 5). * indicates P< 0.01. **(C–E)** Scratch wound-healing assay, colony formation assay, and transwell assay show the inhibition of invasion and migration in the HCC95 cell line (n= 5). **(F)** AURKA, also known as STK6, is prognostic for NSCLC patients’ overall survival. **(G, H)** Protein-ligand docking analysis shows 3-D structure models for AURKA-ginkgolide B and AURKA-triamterene. **(I)** AURKA Lys143 forms a pocket region with ginkgolide B and interacts by hydrogen bonds of Lys258 and Thr292. **(J)** AURKA Arg179 forms a pocket region with triamterene, and interacts by a hydrogen bond of Glu170 and a π-π stacking of Tyr199. The hydrogen bonds were visualized in black dashed lines.

To identify candidate drugs for LUSC, we submitted up-regulated genes from DEGs to the Connectivity Map tool. We identified ginkgolide B and triamterene as the two most significant candidate durgs (P <0.01). Two additional datasets were used to validate the treatment efficacy of the two drugs *in vitro*. We identified differential genes in the two datasets and found an overlap between the three gene lists (**Figure 10A**). Interestingly, top DEGs such as GINS1, AURKA, CDC20, CCNB2, UBE2S, KIF20A, and CDKN3 were among the overlapped genes and significantly down-regulated by the two drugs. As platinum drug is the standard chemo-drug for LUSC patients, we analyzed a dataset of lung samples treated by platinum and found that genes CDC20, CCNB2, and AURKA were also down-regulated (7). Functional enrichment analysis confirmed that the two drugs may target cell cycle related genes. Cell proliferation, invasion, and migration analyses confirmed that the two drugs can decrease the proliferation and invasion of the HCC95 cell line *in vitro* (**Figures 10B–E**). As AURKA is a well-known drug target and is prognostic for overall survival in LUSC (**Figure 10F**), we performed protein-ligand docking analysis to reveal mechanisms. It showed the potential interactions between AURKA and ginkgolide B and triamterene (**Figures 10G, H**). Pose view analysis showed that the interactions may occur at sites Lys258, Thr292, Glu170, and Tyr199 (**Figures 10I, J**).

## Discussion

Precise dissection of tumor tissue transcriptome into the cellular composition is an important method for tumor heterogeneity studies in lung cancer, which has the potential to discover cancer diagnostics and treatment (26). State-of-the-art systems biology methods have been developed for human disease research, such as gene-, module- and cellular-level investigation (27). However, a comprehensive analysis of lung cancer cell subtypes and ecosystems is lacking. Here, we utilized the latest Ecotyper method and showed the contributions of diverse tumor cell subtypes and ecotypes that have been previously ignored to two major lung cancer subtypes LUAD and LUSC.

LUSC generally has a poorer prognosis than LUAD (6). We first used Ecotyper to delineate cell subtypes and ecotypes in LUAD and LUSC. The difference analysis of cell subtype and ratio between the two diseases revealed possible cellular differences (28). For example, CD4.T.cells.2 and CD4.T.cells.4 are naïve and resting T cell subsets. Both T cell subtypes were down-regulated in LUSC compared to LUAD. The favorable roles of higher absolute counts of circulating T cells in NSCLC have been reported (29). Furthermore, several macrophage subpopulations were identified with different expression and prognosis significance. Macrophages.9 and macrophages.1 had similar expression patterns in LUAD, both of which are favorable factors for survival in LUAD but not LUSC, and are down-regulated in LUSC. Macrophages.1 is annotated as monocyte, while macrophages.9 is unknown. These results indicate that more monocytes are recruited to LUAD, which may be a factor for the better prognosis compared to LUSC. Macrophages.2, annotated as classical M0, shows down-regulation in LUSC and favorable prognostic significance in LUAD. Macrophages.5, annotated as M2-like normal macrophage, is a favorable factor in both LUAD and LUSC, displaying lower expression in LUSC. Macrophages.8, annotated as proliferative, shows up-regulation in LUSC but does not exhibit prognostic significance. Macrophages.7, annotated as M2-like proliferative macrophage, shows up-regulation in LUSC and unfavorable prognostic significance in LUAD. Moreover, some of the cell subtypes are novel. For example, Ecotyper provides 5 cell subtypes for epithelial cells, among them epithelial.cells.3 and epithelial.cells.5 had opposite prognosis outcomes. We confirmed the prognostic role of the marker gene of these cell subtypes in an independent dataset (17). For epithelial.cells.5, ARG2 is the top marker gene, which is related to hypoxia-associated renal epithelial cell damage and fibrosis (30). Epithelial.cells.3 is a favorable factor for survival and its top maker gene is ITGA3, which could regulate stemness and epithelial-mesenchymal transition of breast cancer cells (31). Macrophages.1 top maker gene is CCR2, which plays a crucial role in macrophage recruitment (32). Thus, our analysis discovered novel cell subtypes in lung cancer that may serve as biomarkers and merit further experimental validation.

After quantifying the cell subtypes, we investigated the prognostic value of cell state ratios. A well-established cell ratio is the lymphocyte/monocyte ratio, which has been identified to be associated with SCLC and NSCLC survival (33, 34). A high neutrophil/lymphocyte ratio indicated worse overall survival in both SCLC and NSCLC (35). We mathematically identified hundreds of cell state ratios associated with prognosis, and most of them are novel. An example is the Macrophages.1/7 ratio, which is most significant for LUAD survival prognosis across all possible macrophage subpopulation ratios. The prognosis significance of the cell ratio can be validated by the marker gene ratio (**Supplementary Figure 1**). However, these ratios need to be biologically annotated by further experimental validation.

Higher-level tumor ecosystems were also analyzed. We found that different CEs were prognostic in LUAD and LUSC, indicating the different cellular components in the two subtypes. We also found that the ecotype ratios can be prognostic and may result in better predicting performance as the P values are lower than using a single ecotype. Clinically, the cell ratio may have a larger potential to become a practical biomarker as it is unit free and may be comparable across labs and experiments than an absolute gene expression value.

We performed traditional DEG analysis comparing the high and low macrophages.3/PCs.2 ratio samples in LUSC. Among the 10 most significant DEGs, we found that most of the genes are favorable predictors for overall survival in an independent dataset (17). These results may indicate the robustness of Ecotyper. Moreover, five machine learning models were used to predict LUSC. We found several epithelial cell subtypes contributed to the differences between LUSC and LUAD, which may serve as potential cellular targets for disease treatments. Finally, we used up-regulated DEGs for drug screening and found two promising candidates ginkgolide B and triamterene. Independent datasets validated that these drugs can target cancer-related gene expression. Our tumor cell proliferation, invasion, and migration assay also confirmed the anti-tumor roles of the two promising drugs. As AURKA is a promising drugable target (36) and is prognostic for survival, we selected it to reveal potential drug mechanisms by protein-ligand docking and found potential interaction sites. Interestingly, only recently, two studies reported that the two drugs act as potential active anticancer components in NSCLC (37, 38).

In clinical setting immunotherapy and platinum drugs are used for LUSC and LUAD therapy (39). Our findings may provide biomarkers for drug treatment efficacy assessment. As example, macrophages.3/PCs.2 ratio might correlate with immunotherapy. In an independent dataset (40), marker gene based ratio SLAMF8/CXCR4 was associated with progression-free survival of anti-PD-1 immunotherapy NSCLC patients (**Supplementary Figure 2**). However, currently available cohort for immunotherapy is small (n= 21). Larger cohorts are needed to verify the association. For ratio epithelial.cells.1/B.cells.1, we searched literature using their marker genes and found two meta-analyses that reported the roles of KRT6A and TCL1A in anti-PD1 therapy response (41, 42). However, few reports are based on cell ratio. More investigations are needed to confirm these ratio based immune subtypes before they can serve as predictive biomarkers in the treatment of LUSC and LUAD.

It should be acknowledged that *in vitro* cancer cell line assays might not fully replicate the complexities of *in vivo* tumor environments. Additionally, the use of a single cell line (HCC95) may limit the generalizability of the findings. Future studies could involve multiple cell lines with different metastatic potentials and consider additional relevant assays to provide a more comprehensive understanding of the molecular mechanisms underlying the dysregulated immune microenvironment (43).

In sum, this is the first study to demonstrate the clinical relevance of cell subtypes, ecosystems, and their ratios in LUAD and LUSC. Epithelial subtypes are valuable cellular biomarkers for disease diagnosis and treatment. The results may expand our understanding of the cellular organization in NSCLC with implications for disease mechanisms, disease diagnosis, and precision therapies.

## Data availability statement

The dataset analyzed in the study is available at the public database NCI GDC data portal (https://portal.gdc.cancer.gov/) and NCBI GEO (https://www.ncbi.nlm.nih.gov/geo/).

## Ethics statement

Ethical approval was not required for the studies on humans in accordance with the local legislation and institutional requirements because only commercially available established cell lines were used. Ethical approval was not required for the studies on animals in accordance with the local legislation and institutional requirements because only commercially available established cell lines were used.

## Author contributions

XW: Formal analysis, Investigation, Methodology, Writing – original draft, Writing – review & editing. KZ: Data curation, Validation, Visualization, Writing – review & editing. ZH: Conceptualization, Project administration, Supervision, Writing – review & editing.

## Supplementary material

Supplementary Figure 1 Macropahges.1/7 ratio is prognostic for LUAD survival. The prognostic significance of cell ratio was validated by marker gene ratio CCR/CHI3L2. The ratio remained significant after the smoking adjustment.

Supplementary Figure 2 Marker gene based ratio SLAMF8/CXCR4 was associated with progression-free survival of anti-PD-1 immunotherapy NSCLC patients (n= 21).
