# Supplementary material for: In-depth analysis of immune cell landscapes reveals differences between lung adenocarcinoma and lung squamous cell carcinoma
Source: Front Oncol. 2024 Jan 25;14:1338634. doi: 10.3389/fonc.2024.1338634 (PMC10850392; doi:10.3389/fonc.2024.1338634)
Supplement: Supplementary Figure 1 — Macropahges.1/7 ratio is prognostic for LUAD survival. The prognostic significance of cell ratio was validated by marker gene ratio CCR/CHI3L2. The ratio remained significant after the smoking adjustment. [file Image_1.pdf]

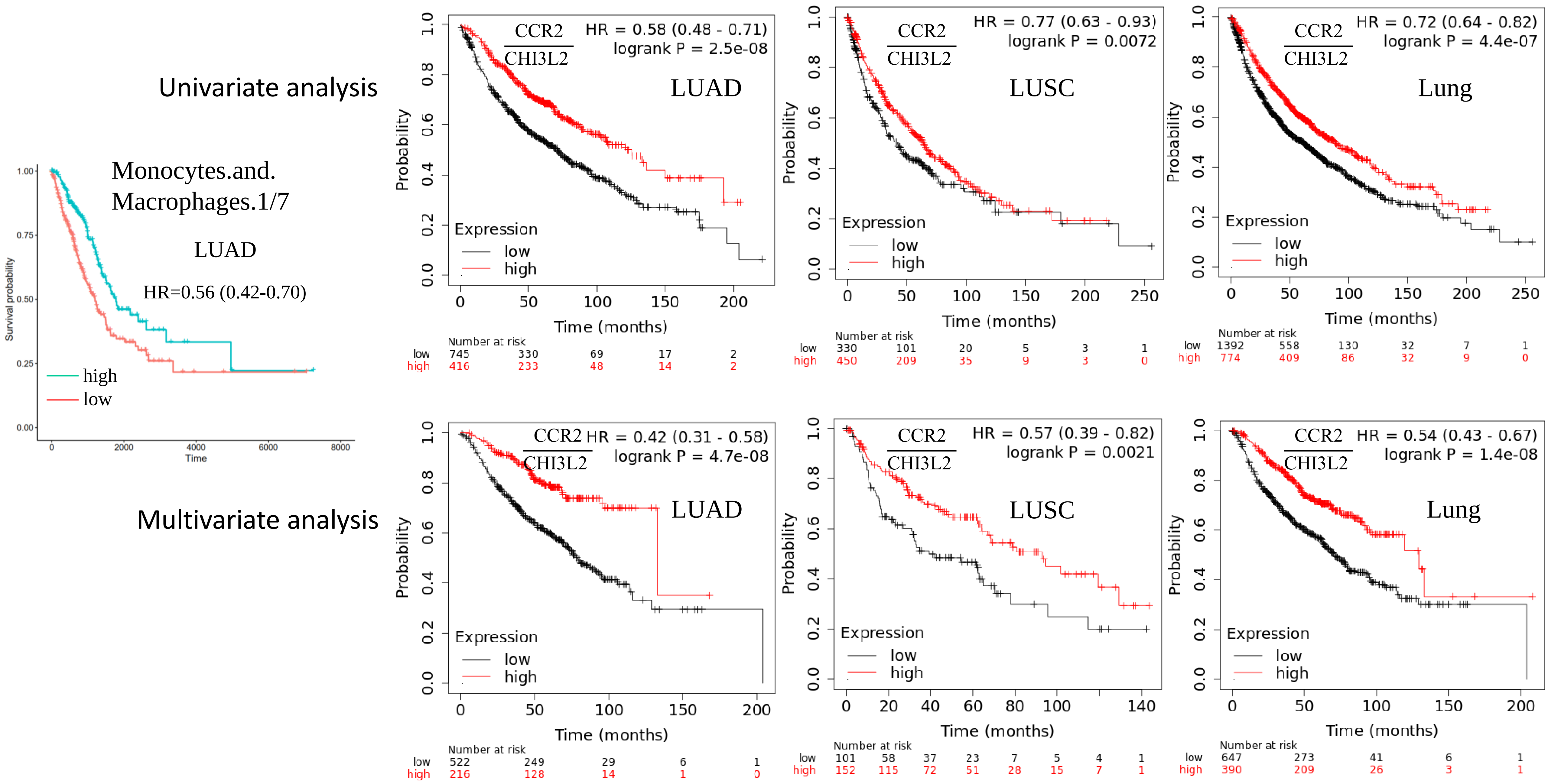

	P value	Hazard Ratio
Smoking history	0.0003	0.55 (0.4 - 0.76)
Selected gene	0	0.47 (0.34 - 0.64)

	P value	Hazard Ratio
Smoking history	0.1949	2.15 (0.68 - 6.85)
Selected gene	0.0019	0.56 (0.39 - 0.81)

	P value	Hazard Ratio
Smoking history	0	0.51 (0.38 - 0.69)
Selected gene	0	0.59 (0.48 - 0.74)
