# Supplementary figures and images for: In-depth analysis of immune cell landscapes reveals differences between lung adenocarcinoma and lung squamous cell carcinoma
Source: Front Oncol. 2024 Jan 25;14:1338634. doi: 10.3389/fonc.2024.1338634 (PMC10850392; doi:10.3389/fonc.2024.1338634)

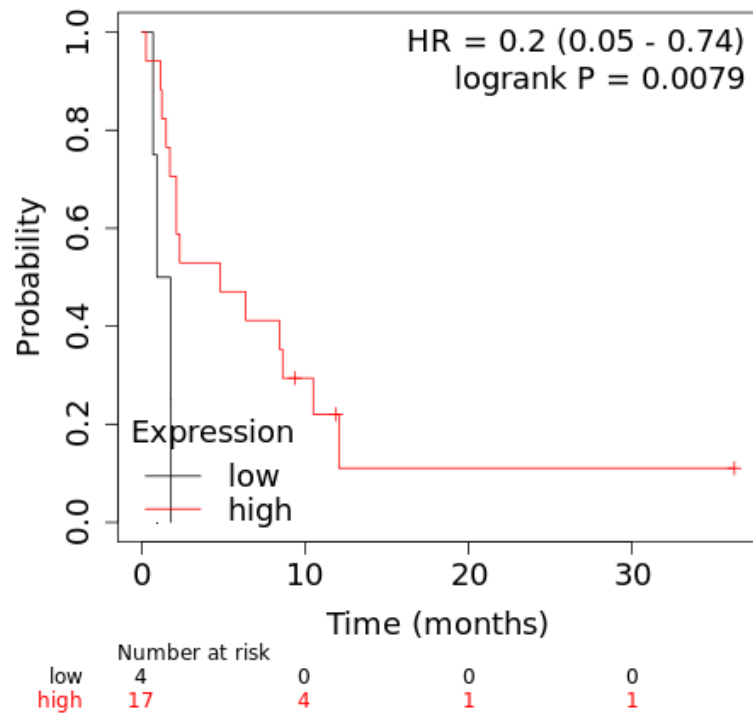

Figure S2

Supplement: Supplementary Figure 2 — Marker gene based ratio SLAMF8/CXCR4 was associated with progression-free survival of anti-PD-1 immunotherapy NSCLC patients (n= 21). [file Image_2.pdf]
